# Early Posttransplant Isolated v1 Lesion Does Not Need to Be Treated and Does Not Lead to Increased Fibrosis

**DOI:** 10.1155/2016/4603014

**Published:** 2016-05-17

**Authors:** Irfan Moinuddin, Bijin Thajudeen, Amy Sussman, Machaiah Madhrira, Erika Bracamonte, Mordecai Popovtzer, Pradeep V. Kadambi

**Affiliations:** ^1^Division of Nephrology, University of Arizona, Tucson, AZ 85724, USA; ^2^Department of Pathology, University of Arizona, Tucson, AZ 85724, USA; ^3^Division of Nephrology, Southern Arizona Veterans Affairs Health Care System, Tucson, AZ 85723, USA

## Abstract

Acute vascular rejection (AVR) is characterized by intimal arteritis in addition to tubulitis and interstitial inflammation. It is associated with a poorer prognosis compared to tubulointerstitial rejection (AIR) and AVR is associated with a higher rate of graft loss than AIR. The prognosis and treatment of arteritis without tubulitis and interstitial inflammation (isolated v1 lesion) are still controversial. We report a case of a patient who had a biopsy of the kidney allograft for evaluation of slow graft function. The biopsy revealed an isolated v1 lesion. However, we chose not to augment immunosuppression. The patient's kidney allograft function improved over time with close monitoring. Repeat biopsy a year later showed no evidence of endothelialitis and relatively unchanged fibrosis and no other abnormalities. Although it is suggested that most cases of isolated v1 lesions will respond to corticosteroids or T cell depleting therapies, some cases will improve with conservative management. Further studies are needed to determine which cases could be managed conservatively.

## 1. Introduction

End stage renal disease (ESRD) is associated with significant cardiovascular morbidity and mortality. When compared to dialysis, kidney transplantation prolongs life and is also associated with improved quality of life. The current graft survival rates at 1, 5, and 10 years are 88%, 77%, and 60%, respectively, on tacrolimus based immunosuppression. However, the incidence of acute rejection is 11.4% over a mean follow-up of 94 months ± 84 months (minimum follow-up of 6 months) [1].

Cell mediated rejection can be classified as AIR and AVR [2]. AVR is usually characterized by the presence of tubulointerstitial inflammation in addition to arteritis. Since 2007, reports of isolated vascular (v1) lesions, characterized by endothelial inflammation with minimal tubulitis and minimal interstitial inflammation, have been increasingly described [3–11]. Banff 2009 guidelines defined isolated v lesions as arteritis with minimal interstitial inflammation (i < 1; 10–25% of parenchymal inflammation) and minimal tubulitis (t < 1; 1–4 mononuclear cells per tubular cross section) [4]. Banff 2011 guidelines redefined isolated v1 lesion as mild to moderate arteritis (v1) with mild interstitial inflammation (i < 2; 26–50% of parenchymal inflammation) and mild tubulitis (t < 2; 5–10 mononuclear cells per tubular cross section) [5].

A proportion of isolated v1 lesions may be associated with donor specific antibodies (DSA), transplant glomerulopathy, arteriosclerosis, glomerulitis, or C4D positivity suggesting that isolated v1 lesions in some cases may represent antibody-mediated rejection. It is not known whether adding anti-B cell/antibody depleting strategies in these cases may prolong graft survival [11, 12].

Incidence of reversal of acute vascular rejection has varied from 75 to 100% in different series [13, 14]. Whether or not to treat isolated v1 lesions remains controversial as there are no randomized controlled trials addressing whether overall benefits of treatment outweigh harm. Our report asks the question of whether isolated v1 lesions should be treated like AVR or if conservative measures may be appropriate in select cases.

## 2. Case Report

Patient is a 70-year-old female with ESRD due to hypertension and obstructive uropathy. She was on hemodialysis for 1 year and received a kidney transplant from a 45-year-old deceased donor. Cold ischemia time was 29 hours and warm ischemia time was 30 minutes. Panel of Reactive Antibodies (PRA) was 0% for both HLA class I and class II antigens. Both the T cell and B cell crossmatches were negative. She was induced with basiliximab and was maintained on mycophenolate mofetil (MMF), tacrolimus, and prednisone. Her postoperative creatinine was 4.98 mg/dL.

Two days postoperatively, she developed atrial fibrillation with rapid ventricular rate, hypotension, and tachypnea and was transferred to intensive care unit, where she was intubated and started on vasopressors. Patient was noted to have leukocytosis in association with diarrhea of unclear etiology. On postoperative day (POD) 4, the patient started to have drainage from the Jackson Pratt drain (placed at the time of transplantation) which was suggestive of small bowel content. The patient was emergently taken to the operating room for repair of bowel leak and a partial small bowel resection was performed. She was started on broad-spectrum antibiotics. Her white count decreased from 23 G/L to 12 G/L. Creatinine plateaued at 3.0 mg/dL. JP drain started having increasing urine output and the fluid creatinine level was 18.8 mg/dL raising the suspicion of urine leak. Renogram confirmed ureteral leak, which was repaired surgically.

**Figure 1:**
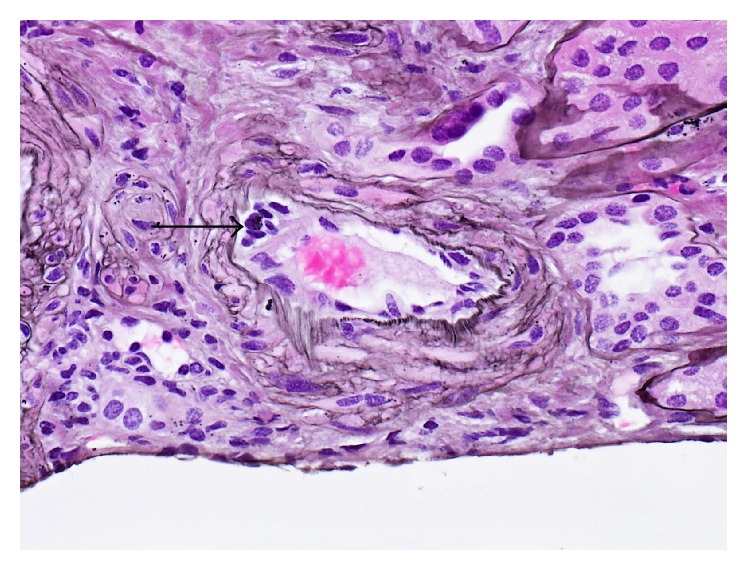
An interlobular artery shows a small collection of lymphocytes beneath the vascular endothelium, sufficient for v1 lesion according to Banff criteria (thick arrow). Jones 400x magnification.

An intraoperative biopsy of the transplanted kidney showed focal endothelialitis with minimal tubulitis (Banff t1) and minimal interstitial inflammation (Banff i0) (Figure 1). The glomeruli showed mild increase in mesangial matrix and slight increase in mesangial cellularity. There was no significant glomerulitis and capillary loops appeared normal, without duplication. No segmental sclerosis was identified. There was a background of mild to moderate patchy interstitial fibrosis with associated focal tubular atrophy. Tubular epithelial cells appeared focally enlarged and reactive; however, no viral change was noted and SV40 immunohistochemical stain was negative. Several tubules showed flattening of the epithelium and loss of brush borders. There was no significant peritubular capillaritis. Two muscular arteries showed endothelialitis characterized by small clusters of mononuclear cells beneath the endothelium. No fibrinoid necrosis or transmural inflammation was seen.

Because of ongoing sepsis, it was decided not to treat her with antilymphocyte therapy. Patient had slow graft function but did not fulfill the diagnosis of delayed graft function because she never required dialysis. Over the next two weeks, her clinical course improved and she was discharged home on POD 41 with creatinine of 0.90 mg/dL. Eleven months later, her creatinine was 1.13 mg/dL.

A repeat biopsy was performed a year after the initial diagnosis of the isolated v1 lesion. The biopsy did not show any evidence of endothelialitis, showed relatively no change in fibrosis, and did not have any other abnormalities.

## 3. Discussion

An isolated v1 lesion is defined as endothelial inflammation (isolated endarteritis) in the absence of significant tubulitis and interstitial inflammation and the natural evolution of this lesion remains unknown [3]. A proportion of isolated v1 lesions may be associated with donor specific antibodies (DSA), transplant glomerulopathy, arteriosclerosis, glomerulitis, or C4D positivity suggesting that isolated v1 lesions in some cases may represent antibody-mediated rejection [11, 12]. However, our biopsy was not in any way characteristic of features of antibody-mediated rejection.

Should isolated v1 lesions be considered as deleterious as T cell mediated rejection or are they more benign? Wu et al. suggested that outcomes associated with isolated v lesions are not more favorable compared to vascular rejection with more severe tubulointerstitial inflammation [6]. However, Shimizu et al. proposed that isolated v lesions represent ischemic changes because they are seen in both compatible and incompatible renal transplantation recipients, seen early after transplantation, and are associated with delayed graft function [7]. Since isolated v1 lesions have not been reported in native kidneys with ischemic injury, these lesions are unlikely to represent a simple ischemic response.

Genomic studies have indicated that isolated v1 lesions may not represent true T cell mediated rejection. This is based on findings of minimal transcriptional activity of T cells, cytokines, and chemokines in kidney transplant biopsies of isolated v1 lesions [8–10]. Salazar et al. used microarray based molecular tests to examine prognosis of v lesions; in their study, most early isolated v lesions had no molecular rejection and overall isolated v lesions in indication biopsy specimen did not affect prognosis [15].

A recent study by Sis et al. retrospectively analyzed whether isolated v1 lesions respond to rejection treatment and whether treatment affects kidney transplant survival. The authors enrolled patients in three groups: isolated endarteritis, acute T cell rejection (positive control), or normal biopsy (negative control). Exclusion criteria included C4d deposition, ABO incompatibility, and crossmatch positivity. Both the isolated endarteritis group and the positive control group were treated with corticosteroids or T cell depleting therapy; negative controls received no treatment. Mean creatinine decrease was 1.5 mg/dL for the endarteritis limb (*p* < 0.001), 1.09 mg/dL for the positive control (*p* = 0.003), and 0.21 mg/dL in the negative control. After rejection treatment functional improvement occurred in 80% of the endarteritis group and in 81% of positive controls. Over a median 3.2-year follow-up, kidney graft survival was 79% in isolated endarteritis, 79% in positive controls, and 91% in negative controls. It was concluded that isolated v1 lesion is an independent risk factor for kidney transplant failure. Furthermore, 20% of patients with isolated v1 lesions did not show functional improvement even after treatment and 17% eventually progressed to graft failure [11].

Some of the deficiencies in the study by Sis et al. include the fact that there was no randomized group in which isolated v1 lesions were managed conservatively. However, 15% of isolated v1 lesions in this study were not treated; the survival difference between untreated isolated v1 lesions and treated isolated v1 lesions was not statistically significant. Furthermore, repeat biopsies at the end of the study period were not performed to determine if isolated v1 lesions had resolved. It would be important to have biopsy evidence to suggest that improvement in renal function was dependent on resolution of isolated v1 lesions or that worsening of renal function was attributable to progression of isolated v1 lesions.

Our patient had an isolated v1 lesion which was not treated because of recent bowel leak and sepsis. She was noted to have a persistent urine leak and was treated initially with ureteral reimplantation and subsequently with nephrostomies. Creatinine subsequently normalized from an initial value of 4.98 mg/dL to 1.13 mg/dL and remained stable for eighteen months after transplant. Repeat biopsy performed a year after the initial diagnosis of isolated v1 lesion did not show any endothelialitis and showed relatively no change in fibrosis. However, the question of whether such patients should be treated for rejection or if they should be followed up with or without periodic biopsies without augmentation of immunosuppression should be addressed with appropriate randomized controlled trials. Whether genomic studies have the capability to differentiate between the aforementioned management options also needs further investigation.
